# Tumor Cell‐Intrinsic CD96 Mediates Chemoresistance and Cancer Stemness by Regulating Mitochondrial Fatty Acid β‐Oxidation

**DOI:** 10.1002/advs.202202956

**Published:** 2022-12-29

**Authors:** Jiang Li, Qidong Xia, Can Di, Chunni Li, Hang Si, Boxuan Zhou, Shubin Yu, Yihong Li, Jingying Huang, Yiwen Lu, Min Huang, Huixin Liang, Xinwei Liu, Qiyi Zhao

**Affiliations:** ^1^ Guangdong Provincial Key Laboratory of Malignant Tumor Epigenetics and Gene Regulation Medical Research Center Sun Yat‐Sen Memorial Hospital, Sun Yat‐Sen University Guangzhou 510120 China; ^2^ Breast Tumor Center Sun Yat‐Sen Memorial Hospital, Sun Yat‐Sen University Guangzhou 510120 China; ^3^ Department of Infectious Diseases Third Affiliated Hospital, Sun Yat‐Sen University Guangzhou 510630 China; ^4^ Guangdong Key Laboratory of Liver Disease Research Third Affiliated Hospital, Sun Yat‐Sen University Guangzhou 510630 China; ^5^ Department of Breast Surgery The First Affiliated Hospital, Zhengzhou University Zhengzhou 450052 China

**Keywords:** cancer stem cells, CD96, chemoresistance, fatty acid *β*‐oxidation, mitochondrial remodeling

## Abstract

Targeting CD96 that originates in immune cells has shown potential for cancer therapy. However, the role of intrinsic CD96 in solid tumor cells remains unknown. Here, it is found that CD96 is frequently expressed in tumor cells from clinical breast cancer samples and is correlated with poor long‐term prognosis in these patients. The CD96^+^ cancer cell subpopulations exhibit features of both breast cancer stem cells and chemoresistance. In vivo inhibition of cancer cell‐intrinsic CD96 enhances the chemotherapeutic response in a patient‐derived tumor xenograft model. Mechanistically, CD96 enhances mitochondrial fatty acid *β*‐oxidation via the CD155‐CD96‐Src‐Stat3‐Opa1 pathway, which subsequently promotes chemoresistance in breast cancer stem cells. A previously unknown role is identified for tumor cell‐intrinsic CD96 and an attractive target in improving the chemotherapeutic response.

## Graphical abstract

CD96 expressed in tumor cells is correlated with poor long‐term prognosis in breast cancer patients. The CD96^+^ cancer cell subpopulations exhibit features of both stemness and chemoresistance. Mechanistically, CD96 enhances mitochondrial fatty acid *β*‐oxidation via the CD155‐CD96‐Src‐Stat3‐Opa1 pathway, which subsequently promotes chemoresistance in breast cancer stem cells.

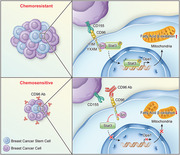

## 1 Introduction

Immunotherapy using immune checkpoint inhibitors has revolutionized anticancer treatment. Immune checkpoint inhibitors for cytotoxic T lymphocyte antigen 4 (CTLA4) or the programmed cell death protein 1 (PD1) pathway have achieved impressive success rates in the treatment of various types of malignancies.^[^ ^1^, ^2^ ^]^ However, the response rates of patients to anti‐CTLA4 and anti‐PD‐1 treatments remain low in many cases, leading to studies of other immune checkpoints.^[^ ^3^, ^4^ ^]^ A better understanding of checkpoint molecules may support the development of next‐generation cancer immunotherapies.

CD96, a novel immune checkpoint protein, together with CD226 and TIGIT form a receptors pathway that closely resembles the CD28/CTLA‐4 pathway.^[^ ^5^ ^]^ CD96 has been shown to function as an important inhibitor of natural killer (NK) and T‐cell activity.^[^ ^6^, ^7^, ^8^ ^]^ Although intracellular signal transduction via CD96 has not been well‐characterized, recent studies have suggested that therapies targeting CD96 can be developed for cancer immunotherapy. Immune checkpoint inhibitors against CD96 improved antitumor immune responses in multiple experimental mouse tumor models.^[^ ^6^, ^9^, ^10^, ^11^ ^]^ In preclinical studies, a greater reduction in the metastatic burden was observed when using a combination of anti‐CD96 monoclonal antibody with either anti‐CTLA‐4 or anti‐PD‐1 monoclonal antibodies, as compared to monotherapy in mouse colon carcinoma, melanoma, and fibrosarcoma models.^[^ ^9^, ^12^ ^]^ In addition to acting as an immune inhibitor, a recent study showed that the CD96 gene index was significantly associated with biochemical recurrence‐free survival in patients with prostate cancer undergoing radical radiotherapy.^[^ ^13^ ^]^

Expression of CD96 is limited to immune cells and has been reported primarily on NK and T cells. Additionally, CD96 is highly expressed in T‐cell acute lymphoblastic leukemia and has been reported as a cancer stem cell marker in acute myeloid leukemia.^[^ ^14^ ^]^ Nevertheless, whether CD96 is expressed in solid tumor cells remains unknown. Here, we evaluated the CD96^+^ cell subpopulations among human breast cancer (BC) cells and explored their function and underlying molecular mechanisms of action to explore new targets for tumor therapy.

## 2 Results

### 2.1 CD96^+^ Cancer Cell Subsets Are Associated with Poor Prognosis and Chemoresistance in Patients with BC

**Figure 1:**
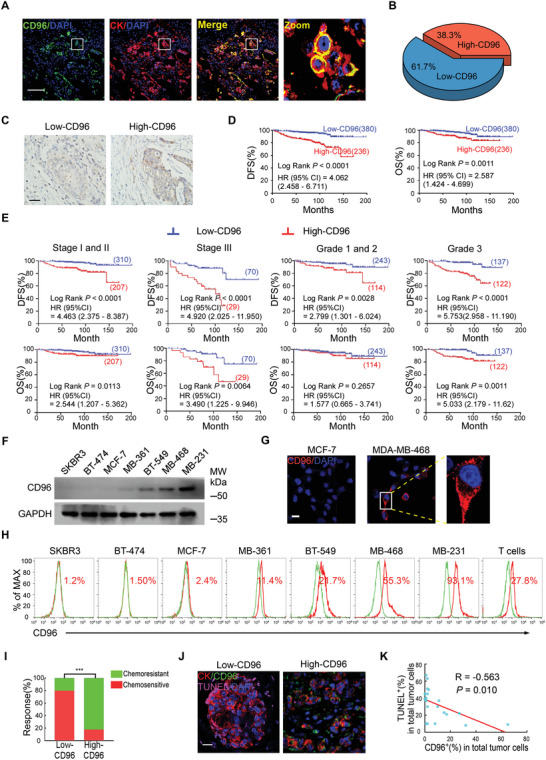
CD96^+^ cancer cell subsets are associated with poor prognosis and chemoresistance in patients with breast cancer. A) Representative immunofluorescence staining of CD96 (green) and CK (red) in breast cancer samples. Nuclei were counterstained with DAPI (blue). Scale bar, 100 µm. B) The proportion of CD96 expression in 616 breast cancer samples based on immunoreactive score, the high and low expression was divided by X‐tile statistical software. C) Representative immunohistochemistry images of CD96 protein in clinical breast cancer tissues. Scale bar, 50 µm. D) Kaplan–Meier survival curves of patients with breast cancer containing low and high infiltration numbers of CD96^+^ cancer cells, disease‐free survival (DFS) and overall survival (OS) are shown (*n* = 616). E) Kaplan–Meier survival curves for patients with breast cancer containing low and high CD96 abundance in different tumor staging (stage I/II and stage III) and histological grading (grade 1/2 and grade 3) of breast cancers (*n* = 616). F) CD96 expression was evaluated by western blotting. Representative image is shown from 3 independent experiments. G) Representative immunofluorescence staining of CD96 protein (red) in MCF‐7 and MDA‐MB‐468. Nuclei were counterstained with DAPI (blue). Scale bars, 20 µm. H) Representative flow cytometry histogram of CD96 expression on indicated breast cancer cell lines, human peripheral blood T cell as a positive control. I) The chemotherapeutic responses were evaluated using the RECIST standard in breast cancer patients with low or high tumoral CD96. ***p < 0.001 by Fisher's exact test, *n* = 337 for low CD96 and *n* = 203 for high CD96. J) Representative immunofluorescence images for TUNEL^+^CK^+^ apoptotic tumor cells in patients with breast cancer after neoadjuvant treatment. Scale bars, 20 µm. K) The correlation between the percentage of CD96^+^ and TUNEL^+^ tumor cells in breast cancer samples was analyzed via Spearman statistic (*n* = 20).

CD96 is reportedly enriched in tumor‐infiltrating T cells and NK cells in various cancers.^[^ ^14^, ^15^ ^]^ However, the role of CD96 in solid tumor cells is hardly known.^[^ ^16^ ^]^ Thus, we examined the expression pattern of CD96 in solid tumors using co‐immunofluorescence‐mediated evaluation of CD96 and various cell specific markers across 616 clinical samples from patients with BC (Table S1, Supporting Information). Interestingly, a fraction of cytokeratin (CK)^+^ tumor cells were strongly positive for CD96 (**Figure** **1**A). CD96 staining was also detected in tumor‐infiltrating CD8^+^ T cells but was only minimally detected in CD31^+^ endothelial cells or *α*‐SMA^+^ fibroblasts in patients with BC (Figure S1A, Supporting Information), which is consistent with the results of previous studies.^[^ ^17^ ^]^ Importantly, the application of optimal cutoff points, as determined using X‐tile statistical software, revealed that 38.3% of patients with BC expressed high levels of CD96 (Figure 1B,C), and that such high tumoral CD96 expression was associated with worse disease‐free survival (DFS) and overall survival (OS) (Figure 1D). Stratified analysis revealed that patients with high tumoral CD96 expression experienced shorter disease‐free survival and overall survival than those with low tumoral CD96 expression in most subgroups (Figure 1E and Figure S1B, Supporting Information). Multivariate Cox regression analysis also revealed that tumoral CD96 was an independent prognostic factor for OS and DFS after adjusting for other prognostic variables (Tables S2 and S3, Supporting Information). We confirmed that CD96 was expressed in multiple BC cell lines using western blotting and observed diverse CD96 expression in various BC cell lines (Figure 1F). Immunofluorescence staining confirmed that a considerable fraction of MDA‐MB‐468 cells expressed CD96 (Figure 1G), whereas relatively few MCF‐7 cells were positive for CD96. Quantification by flow cytometry revealed that surface CD96 expression ranged from 1.8 ± 0.6% to 92.7 ± 2.7% in various BC cell lines (Figure 1H and Figure S1C, Supporting Information).

Subsequently, we evaluated the efficacy of chemotherapy in patients with BC using the Response Evaluation Criteria in Solid Tumors (RECIST). We found that patients with BC containing low CD96 levels had better therapeutic outcomes than those with high CD96 levels (Figure 1I). Moreover, tumors with high CD96 expression contained fewer apoptotic cells than those with low CD96 expression in patients with BC after neoadjuvant treatment (Figure 1J). The percentage of cancer cell‐intrinsic CD96 was inversely associated with the percentage of apoptotic tumor cells in patients with BC who underwent neoadjuvant therapy (Figure 1K). These data indicate that human BCs contain a subpopulation of CD96^+^ tumor cells, which correlates with poor prognosis and chemoresistance.

### 2.2 Cancer Cell‐Intrinsic CD96 Promotes Tumor Progression and Chemoresistance in Patient‐Derived Tumor Xenografts In Vivo

**Figure 2:**
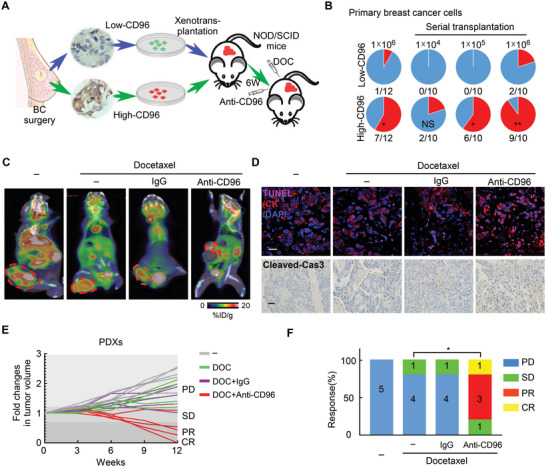
Cancer cell‐intrinsic CD96 promotes tumor progression and chemoresistance in patient‐derived tumor xenografts in vivo. A) Experiment schematic: tumor cells with low or high CD96 expression from clinical samples of patients with breast cancer were transplanted into fat pads of NOD/SCID mice to establish PDXs, which were treated with docetaxel and CD96 blocking antibody. B) Incidence of tumorigenesis in primary breast cancer cells with low or high CD96 expression was evaluated using serial transplantation. NS > 0.05; *p < 0.05; **p < 0.01 compared to primary breast cancer cells with low CD96 expression by Fisher's exact test. C) Representative images of tumor growth capacity in PDXs with high CD96 expression was monitored by PET‐CT after treatment as indicated reagents. The circles indicate PDXs tumor. D) Representative immunofluorescence images for TUNEL^+^CK^+^ apoptotic tumor cells (up) and immunohistochemistry for Cleaved‐Cas3 (bottom) in PDXs with high CD96 expression, mice were treated as (A). Scale bars, 20 µm (up) and 100 µm (down). E,F) PDXs were treated as (A). The fold changes of tumor size (E) and therapeutic responses were evaluated using the RECIST standard (F). *p < 0.05 compared to the docetaxel alone by Fisher's exact test, *n* = 5 per group.

To investigate the role of cancer cell‐intrinsic CD96 in tumor progression and drug resistance in vivo, we established a patient‐derived tumor xenograft (PDX) model of BC in immunocompromised mice. Tumor cells obtained during surgical operation on tumors showing high‐CD96 or low‐CD96 expression were transplanted into NOD/SCID mice, respectively (**Figure** **2**A). Tumorigenicity was much stronger in immunocompromised mice injected with high‐CD96 primary BC cells than in those injected with low‐96 primary BC cells (Figure 2B). As a novel immune checkpoint receptor in T cells and NK cells, accumulating data support that targeting CD96 can improve the anti‐tumor immune response.^[^ ^8^, ^10^, ^12^, ^18^ ^]^ We therefore examined whether blocking tumoral CD96 could improve cancer therapy in PDX models of immunocompromised mice (Figure 2A). The combination of docetaxel and a CD96 blocking antibody dramatically reduced tumor growth and enhanced apoptosis of tumor cells in high‐CD96 expressing PDXs (Figure 2C,D). Moreover, therapeutic efficiency was evaluated using the RECIST standard, which revealed that addition of a CD96 blocking antibody substantially enhanced the chemotherapeutic response of these PDXs (Figure 2E,F). These results indicate that tumor cell‐intrinsic CD96 can be used as a target for CD96 immunotherapy to enhance the effects of chemotherapy in vivo.

### 2.3 CD96 Is Highly Expressed in Breast Cancer Stem Cells

**Figure 3:**
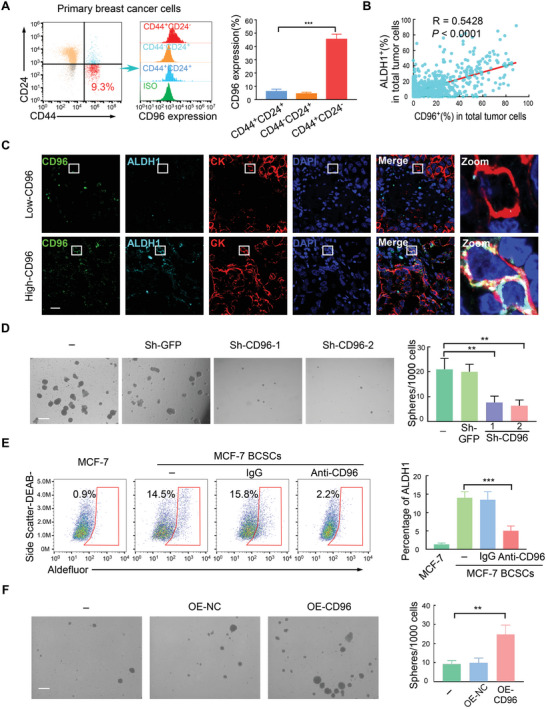
CD96 is highly expressed in BCSCs. A) Primary tumor cells were isolated from human breast cancer samples by CD326 (EpCAM) MicroBeads. CD96 was checked by flow cytometry in stem cell‐like group (CD44^+^CD24^−^) and non‐stem cell group (CD44^−^CD24^+^ and CD44^+^CD24^+^). ***p < 0.001 by One‐Way ANOVA with Tukey's multiple comparisons test (*n* = 5). B,C) Immunofluorescence triple‐staining of clinical breast cancer biopsy for co‐expression of CD96 (green), ALDH1 (pale green), and CK (red). Nuclei were counterstained with DAPI (blue). Scale bar, 20 µm. B) is the correlation between the percentage of CD96^+^CK^+^ and ALDH1^+^CK^+^ cells and C) representative images are shown, *n* = 616. D) SKBR3 BCSCs were transduced with shRNA against CD96, representative images of sphere formation and the quantification are shown. 4 fields per samples; Mean ± SD; **p < 0.01 by One‐Way ANOVA with Tukey's multiple comparisons test (*n* = 3). Scale bar, 200 µm. E) ALDH1 expression in MCF‐7 BCSCs was detected by flow cytometry after treatment with CD96 blocking antibody. Mean ± SD; ***p < 0.001 by One‐Way ANOVA with Tukey's multiple comparisons test, *n* = 4. F) Representative images of mammosphere forming in MCF‐7 with CD96 overexpression. The quantification of sphere formation in left. 4 fields per samples; Mean ± SD; ***p < 0.001 by One‐Way ANOVA with Tukey's multiple comparisons test, *n* = 3. Scale bar, 200 µm.

Breast cancer stem cells (BCSCs) account for a small fraction of cancer cells but play a major role in tumor progression and chemoresistance.^[^ ^19^, ^20^ ^]^ Although CD96 has been reported as a cancer stem cell (CSC) marker in leukemia,^[^ ^15^, ^16^, ^21^ ^]^ the role of CD96 in stemness in solid tumor cells remains unknown. Thus, we evaluated the expression of CD96 on CSCs in primary tumor cells from human BC samples using flow cytometry (**Figure** **3**A). CD96 expression was much higher in CSCs (CD44^+^CD24^−^) than in non‐CSCs (CD44^+^CD24^+^ and CD44^−^CD24^+^) (Figure 3A). Multiple immunofluorescent staining of CD96, ALDH1, and CK demonstrated that CD96 was co‐expressed in most ALDH1^+^CK^+^ cells, and the percentages of CD96^+^ were significantly associated with ALDH1^+^ percentages in CK^+^ tumor cells from clinical BC samples (Figure 3B,C). To validate these clinical findings in vitro, we enriched the BCSC fraction in BC cell lines with low basal CD96 levels (MCF‐7 and SKBR3) on ultra‐low adhesion plates.^[^ ^22^, ^23^ ^]^ Western blot analysis revealed that the CD96 protein levels were significantly increased in BCSCs compared to those in adherent BC cells (Figure S2A, Supporting Information). Taken together, these data suggest that CD96 expression is increased in BCSCs.

We next investigated the role of CD96 in these BCSCs. BCSCs were enriched by prolonged mammosphere culture as we^[^ ^23^ ^]^ and others^[^ ^24^, ^25^ ^]^ described previously. The cells were then transduced with two different short hairpin (sh) RNAs against CD96 (Figure S2B, Supporting Information). This knockdown resulted in a significant reduction in the number of mammospheres (Figure 3D). Consistently, the proportion of ALDH1^+^ cells was dramatically reduced following treatment with blocking antibodies against CD96 (Figure 3E). In contrast, forced CD96 expression enhanced mammosphere formation (Figure 3F and Figure S2C, Supporting Information). These data suggest that CD96 is preferentially expressed in BCSCs.

### 2.4 Expression of CD96 in BCSCs Enhanced Chemoresistance and Promoted Tumor Progression

**Figure 4:**
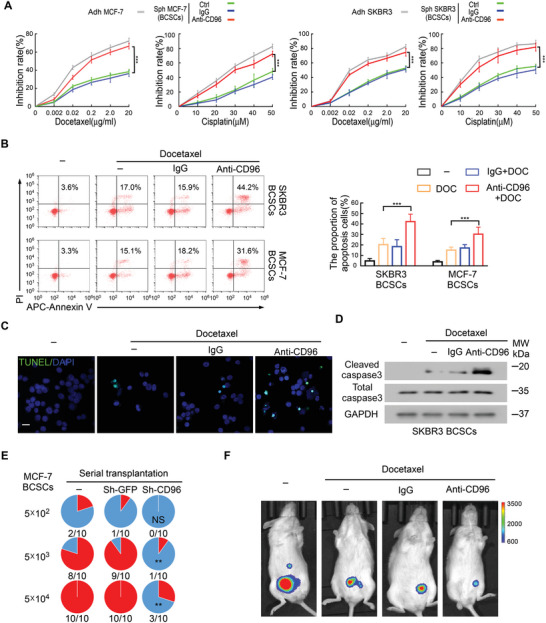
Expression of CD96 in BCSCs enhances chemoresistance and promotes tumor progression. A) MCF‐7 and SKBR3 BCSCs treated with the combination of docetaxel or cisplatin with CD96 blocking antibody, the growth inhibition rate was checked by MTT assay. Mean ± SD; ***p < 0.001 by One‐Way ANOVA with Tukey's multiple comparisons test (*n* = 4). B) SKBR3 and MCF‐7 BCSCs were treated with docetaxel in the presence and absence of CD96 blocking antibody. The proportion of Annexin V^+^/PI^−^ (early apoptosis) and Annexin V^+^/PI^+^ (late apoptosis) cells were detected by flow cytometry. The quantification is shown (right). Mean ± SD; ***p < 0.001 by One‐Way ANOVA with Tukey's multiple comparisons test, *n* = 7. C) The apoptotic rates of MCF‐7 BCSCs were evaluated by TUNEL staining (green), *n* = 3. Scale bar, 20 µm. D) SKBR3 BCSCs were treated with docetaxel in the presence and absence of CD96 blocking antibody, the expression of cleaved/total caspase3 was checked by western blot in 3 independent experiments. E) Incidences of tumorigenesis of the secondary tumor in serial transplantation models. MCF‐7 BCSCs with or without CD96 knockdown were injected into the mammary fat pads of NOD/SCID mice. The xenografts were harvested 6 weeks later, and tumor cells were isolated by CD326 (EpCAM) MicroBeads and serially transplanted into NOD/SCID mice alone. NS > 0.05; **p < 0.01 by Fisher's exact test. F) BCSCs were enriched from luciferase‐expressing MCF‐7 and then transplanted into NOD/SCID mice, docetaxel and CD96 blocking antibody were injected intraperitoneally when tumor was palpable. Representative bioluminescence images in NOD/SCID mice are shown, *n* = 5 per group.

Chemoresistance is a primary factor associated with poor prognosis in patients with cancer and is closely associated with specific features of the CSCs in these patients.^[^ ^22^, ^26^, ^27^ ^]^ To determine whether CD96 participates in chemoresistance in BCSCs, we enriched MCF‐7 and SKBR3 BCSCs and treated them with docetaxel or cisplatin in the presence or absence of a CD96‐blocking antibody in vitro. The inhibition rates of BCSCs treated with docetaxel or cisplatin was dramatically increased when chemotherapy was combined with CD96‐blocking antibody treatment (**Figure** **4**A) or CD96 knockdown (Figure S3A, Supporting Information). This result was supported by the observation that a CD96‐blocking antibody or CD96 knockdown effectively promoted chemotherapy‐induced apoptosis in BCSCs (Figure 4B and Figure S3B, Supporting Information). In addition, both immunofluorescent TUNEL staining (Figure 4C) and western blotting for cleaved caspase3 (Figure 4D) supported these results, demonstrating that treatment with a CD96 inhibitor can potentiate the effects of chemotherapy in BCSCs.

To validate that CD96 enhances chemoresistance in BCSCs in vivo, we constructed a tumor xenograft model using BCSCs with or without CD96 knockdown.^[^ ^22^ ^]^ The results showed that CD96 knockdown reduced tumorigenicity after serial transplantation (Figure 4E), confirming the results of our in vitro experiments (Figure S3A,B, Supporting Information). Interestingly, the combination of CD96‐blocking antibody and docetaxel significantly reduced tumor growth in models with MCF‐7 BCSCs expressing high levels of CD96 (Figures 4F and Figure S3C, Supporting Information), with limited tumor growth in MCF‐7 cells expressing low levels of CD96 (Figure S3D, Supporting Information). Taken together, these results indicate that CD96 enhances chemoresistance in BCSCs and promotes tumor progression.

### 2.5 CD96 Regulates BCSCs Chemoresistance via Regulating Mitochondrial Fatty Acid *β*‐Oxidation

**Figure 5:**
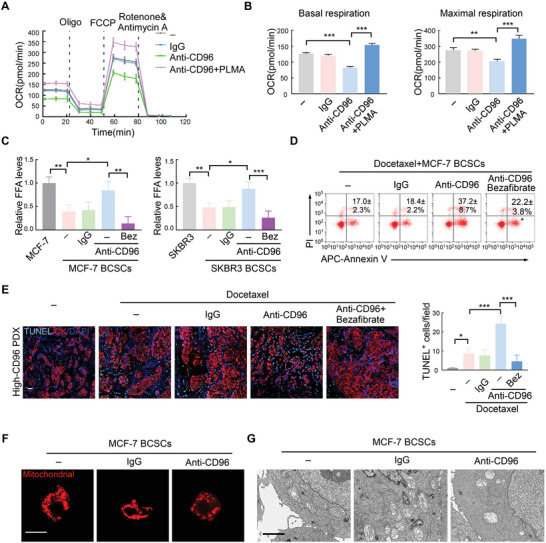
CD96 regulates mitochondrial FAO via maintaining mitochondrial membrane in BCSCs. A) Time series of oxygen consumption rate (OCR) was measured by Seahorse Metabolic Analyzer in MCF‐7 BCSCs, which were treated with CD96 blocking antibody with or without PLMA (palmitate). Data are represented as Mean ± SD. B) Basal and maximal respiration from the samples analyzed in (A). ***p < 0.001 by One‐Way ANOVA with Tukey's multiple comparisons test (*n* = 3). C) FFA levels were quantified in MCF‐7 BCSCs or SKBR3 BCSCs pretreated with IgG or CD96 blocking antibody using Free Fatty Acid Quantification Assay Kit, the relative levels is shown. In some experiments, 5 µm Bezafibrate (Bez, FAO agonists) was added into media with CD96 blocking antibody for 24 h, Mean ± SD; *p < 0.05; **p < 0.01; ***p < 0.001 by One‐Way ANOVA with Tukey's multiple comparisons test (*n* = 3). D) MCF‐7 BCSCs were treated with docetaxel with or without CD96 blocking antibody. FAO agonists were used in some experiments. The proportion of Annexin V^+^/PI^−^ (early apoptosis) and Annexin V^+^/PI^+^ (late apoptosis) cells were detected by flow cytometry. The representative plots and quantification are shown. Mean ± SD; *p < 0.05 compared to third group by One‐Way ANOVA with Tukey's multiple comparisons test (*n* = 3). E) Representative immunofluorescence images for TUNEL^+^CK^+^ apoptotic tumor cells in the harvested PDXs treated as indicated reagents. The quantification of apoptotic is shown in right. Mean ± SD; **p < 0.01; ***p < 0.001 by One‐Way ANOVA with Tukey's multiple comparisons test (*n* = 5). Scale bar, 20 µm. F) MCF‐7 BCSCs were treated with CD96 neutralizing antibody. Representative immunofluorescence images for Mito‐Tracker Red are shown from 3 independent experiments. Scale bar, 10 µm. G) Representative images of transmission electron microscopy in MCF‐7 BCSCs with or without CD96 blocking antibody treatment, *n* = 3; Scale bar = 500 nm.

A hypermetabolic state is indispensable for sustaining CSCs characteristics.^[^ ^28^, ^29^, ^30^ ^]^ Fatty acid *β*‐oxidation (FAO), a crucial metabolic pathway for maintaining the stemness of cancer cells,^[^ ^31^, ^32^, ^33^ ^]^ plays an important role in the chemoresistance of CSCs.^[^ ^27^, ^30^, ^34^ ^]^ Based on this information, we examined whether CD96 is involved in FAO regulation in BCSCs. We first evaluated oxidative phosphorylation (OXPHOS) activity, a metabolic process associated with the utilization of FAO substrates, by measuring the oxygen consumption rate (OCR, an indicator of OXPHOS).^[^ ^35^, ^36^ ^]^ Interestingly, CD96 promoted the effective utilization of fatty acids by BCSCs (**Figure** **5**A,B). Furthermore, the addition of PLMA (poly (lauryl methacrylate), a fatty acid substrate) to the BCSCs rescued the decreased basal and maximum respiration rates downregulated by anti‐CD96 antibodies (Figure 5A,B). Moreover, treatment with a CD96‐blocking antibody exacerbated the over‐accumulation of free fatty acids (FFA), which was rescued by the addition of an FAO agonist (bezafibrate) (Figure 5C). We then evaluated whether this FAO agonist could influence the chemotherapy efficiency improved by anti‐CD96 antibodies. As expected, the addition of bezafibrate reversed the effect of anti‐CD96 antibodies in both in vitro cell culture of BCSCs and in the high‐CD96 PDX model treated with docetaxel (Figure 5D,E; Figure S4A, Supporting Information).

Mitochondrial membrane remodeling and maintenance is crucial for FAO metabolism.^[^ ^37^, ^38^ ^]^ Interestingly, mitochondria fragmentation was observed after CD96 inhibition in BCSCs using Mito‐Tracker and transmission electron microscopy (Figure 5F,G). We then examined the expression of genes associated with the regulation of mitochondrial membrane dynamics (Opa1, Mfn1/2, Drp1 and Mff). Opa1 protein and mRNA levels were markedly reduced in BCSCs treated with CD96‐blocking antibodies (Figure S4B,C, Supporting Information), whereas Mfn1/2, Drp1, and Mff levels were not reduced (Figure S4D, Supporting Information). When mitochondrial fission was inhibited using mitochondrial division inhibitor (Mdivi), the efficiency of chemotherapy induced‐apoptosis by anti‐CD96 was partially reduced (Figure S4E, Supporting Information). Opa1 knockdown significantly enhanced the effect of chemotherapy (Figure S4F,G, Supporting Information), similar to the effects of the CD96‐blocking antibody, suggesting that mitochondrial membrane remodeling is required for CD96‐related chemoresistance. These data suggest that CD96 potentiates mitochondrial fatty acid *β*‐oxidation via maintaining mitochondrial membrane integrity of BCSCs.

### 2.6 CD96 Regulates Mitochondrial FAO via the Src‐Stat3 Pathway in BCSCs

**Figure 6:**
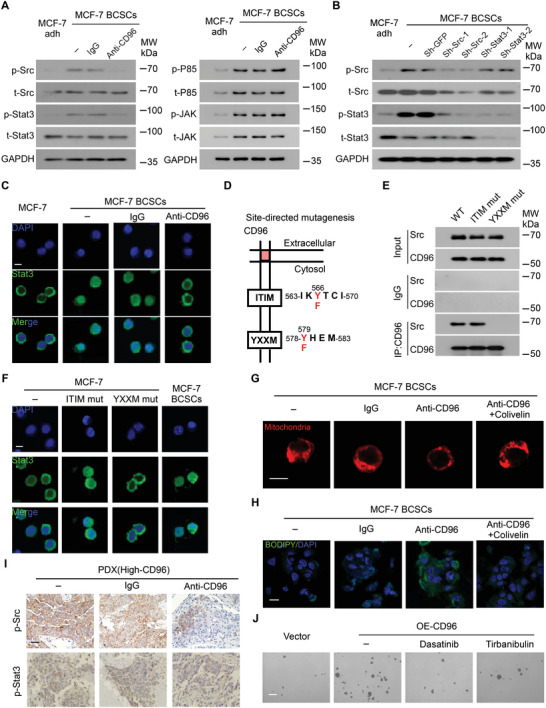
CD96 regulates mitochondrial FAO via the Src‐Stat3 pathway in BCSCs. A) MCF‐7 BCSCs were co‐cultured with CD96 blocking antibody or IgG, total and phosphorylation of Src, Stat3, P85, and JAK were assessed by western blotting. Representative blot is shown from 3 independent experiments, MCF‐7 adherent cells (Adh) as a control. B) Src or Stat3 were silenced by shRNA in MCF‐7 BCSCs, the total and phosphorylation of Stat3 and Src were detected by western blotting. Representative image is shown from 3 independent experiments. C) Immunofluorescent staining for Stat3 (green) in MCF‐7 adherent cells and BCSCs, which were treated with or without CD96 blocking antibody. The experiment was performed twice with similar results. Scale bar, 10 µm. D,E) The CD96 binding sites on Src was checked using co‐IP assays. The pattern of YXXM and ITIM motif mutations (D). Mutations of YXXM binding sites disrupt CD96 co‐IP with native Src but not ITIM (E). F) MCF‐7 cells were transfected with CD96 expression vector with ITIM or YXXM mutation, “−” represents no treatment. Immunofluorescent staining for Stat3 (green) is shown, MCF‐7 BCSCs as a control. The experiment was performed twice with similar results. Scale bar, 10 µm. G,H) MCF‐7 BCSCs were treated with CD96 blocking antibody with or without Stat3 promoter (Colivelin). Representative immunofluorescence images for Mito‐Tracker Red (G) and BODIPY staining (H) are shown from 3 independent experiments. Scale bars, 10 µm for (G) and 20 µm for (H). I) PDXs of breast cancer with high CD96 expression were treated with CD96 blocking antibody, phosphorylation of Src and Stat3 proteins were detected by immunohistochemistry. Scale bar, 100 µm. J) The mammosphere forming of MCF‐7 cells with CD96 overexpression in the presence of Src inhibitor Dasatinib or Tirbanibulin. Scale bar, 200 µm (*n* = 3).

CD96 contains the immunoreceptor tyrosine‐based inhibitory (ITIM) motif and Tyr‐xx‐Met (YXXM) motif, albeit the intracellular signal transduction has not been well characterized.^[^ ^16^, ^18^, ^39^ ^]^ YXXM interacts with signal transduction proteins containing the SH2 domain,^[^ ^11^, ^16^ ^]^ which is crucial for the stemness of CSCs.^[^ ^40^, ^41^ ^]^ Based on this information, we asked whether the CD96 YXXM motif is responsible for BCSCs regulation. We first examined the expression and phosphorylation of a series of SH2‐containing proteins known to participate in CSCs regulation.^[^ ^27^, ^42^, ^43^, ^44^, ^45^, ^46^, ^47^, ^48^ ^]^ Among them, the phosphorylation levels of Src (p‐Src) and Stat3 (p‐Stat3) were markedly decreased following treatment with anti‐CD96 antibodies but not P85 or JAK (**Figure** **6**A). These results suggest that CD96 regulates BCSCs via Src and Stat3. Previous studies suggested that Stat3 is a downstream protein in the Src pathway.^[^ ^48^ ^]^ We tested this hypothesis by creating a set of shRNAs designed to inhibit Src and Stat3 expression (Figure S5A,B, Supporting Information). Transduction with these constructs revealed that the levels of phosphorylated Stat3 were decreased following Src knockdown; in contrast, Stat3 knockdown did not influence the expression of phosphorylated Src (Figure 6B), suggesting that Stat3 is downstream of the Src protein. Furthermore, blocking CD96 abrogated the nuclear translocation of Stat3 in BCSCs (Figure 6C), further suggesting that CD96 regulates Stat3 phosphorylation and nuclear translocation. We validated this CD96/Src interaction using point mutants of the YXXM/ITIM domains in CD96 (Figure 6D). Mutations in the YXXM domain, rather than in the ITIM domain, inhibited CD96/Src binding (Figure 6E). In addition, MCF‐7 cells with YXXM mutations exhibited reduced mammosphere production (Figure S5C, Supporting Information), indicating that CD96 directly binds to the SH2 motif of Src via the YXXM motif and subsequently regulates the stemness of BCSCs. This YXXM mutation also abolished Stat3 nuclear translocation (Figure 6F), revealing that CD96 regulates Stat3 phosphorylation via the YXXM motif.

We then investigated whether the Src‐Stat3 pathway is involved in mitochondrial FAO‐mediated chemoresistance. In BCSCs treated with a CD96 antibody in vitro, the addition of a Stat3 activator (colivelin) rescued the mitochondria fragmentation (Figure 6G) and Opa1, a mitochondrial membrane remodeling protein (Figure S5D, Supporting Information), suggesting that Stat3 signaling is involved in CD96‐related mitochondrial membrane remodeling. In the same BCSCs culture in vitro, FFA over‐accumulation exacerbated by the CD96 antibody (Figure 6H) was rescued by the addition of colivelin, indicating that Stat3 participates in CD96‐dependent FAO metabolism. In vivo, Src and Stat3 phosphorylation was decreased in high‐CD96 PDXs following treatment with CD96 antibodies but not control IgG (Figure 6I). Moreover, Src inhibition by dasatinib or tirbanibulin disrupted mammosphere formation in CD96‐overexpressing tumor cells (Figure 6J and Figure S5E, Supporting Information) and Stat3 phosphorylation in BCSCs (Figure S5F, Supporting Information), supporting that CD96 can sustain cancer cell stemness via the Src‐Stat3 pathway. Taken together, these results indicate that mitochondrial FAO‐mediated chemoresistance in BCSCs is regulated by CD96 via the YXXM‐Src‐Stat3 pathway.

### 2.7 CD96‐Mediated Chemoresistance in BCSCs Requires CD155

**Figure 7:**
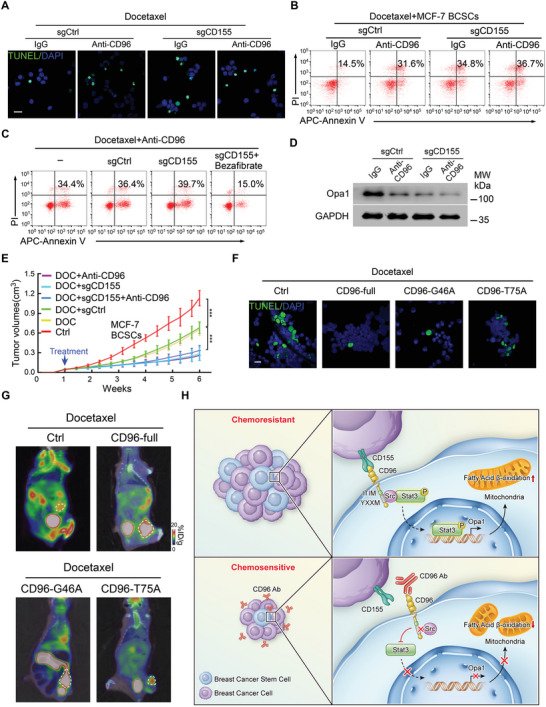
CD96 mediated chemoresistance in BCSCs requires CD155. A–C) MCF‐7 BCSCs with or without CD155 knockout were treated with docetaxel in the presence and absence of CD96 blocking antibody, Bezafibrate was used in some experiments. Representative immunofluorescence images for TUNEL^+^ apoptotic cells (A) and flow cytometry plots for apoptotic cells (B,C) are shown, *n* = 3. Scale bar, 20 µm. D) MCF‐7 BCSCs with or without CD155 knockout were treated with CD96 blocking antibody, representative western blotting image of Opa1 expression is shown from 3 independent experiments. E) MCF‐7 BCSCs with or without CD155 knockout were transplanted into NOD/SCID mice. Docetaxel and CD96 blocking antibody were injected intraperitoneally when tumor was palpable. Tumor size was evaluated every 3 days. ***p < 0.001 by One‐Way ANOVA with Tukey's multiple comparisons test at week 6 (*n* = 6 per group). F,G) MCF‐7 cells infected with CD96‐full and indicated mutation plasmid and treated docetaxel in vitro and vivo. The apoptotic rate was evaluated by TUNEL staining (green) (F) and tumor in fat pad of NOD/SCID mice was assessed by PET‐CT (G), circles indicate tumors, representative images are shown. Scale bar, 20 µm. H) The mechanism diagram of CD96 and CD155 interaction, which regulates cancer stem cell chemoresistance via the Src‐Stat3‐Opa1‐mitochondrial membrane remodeling‐FAO pathway.

CD96 is known to interact with its ligands, CD155 and CD111.^[^ ^49^ ^]^ While CD96 binding to CD111 is predominantly observed in mice,^[^ ^16^ ^]^ human CD96 is selective toward CD155 with a binding affinity even stronger than that of the CD155:TIGHT interaction. However, whether CD155 binding to cancer cell‐intrinsic CD96 directly participates in chemoresistance in solid tumors remains unknown. We first confirmed the expression of CD155 in tumor cells using flow cytometry. CD155 was constitutively and highly expressed in both adherent BC and BCSCs (Figure S6A, Supporting Information), which was effectively knocked out by sgRNA targeting CD155 (Figure S6B, Supporting Information). CD155 knockout promoted chemosensitivity to docetaxel in BCSCs expressing high levels of CD96 (Figure S6C, Supporting Information) but had little effect on BC cells with low CD96 expression (Figure S6D, Supporting Information). Interestingly, blocking of CD96 did not enhance the chemosensitivity to docetaxel in CD155 knockout BCSCs (**Figure** **7**A,B; Figure S6E, Supporting Information). Furthermore, the FAO agonist simultaneously suppressed the pro‐chemotherapy effects of CD155 knockout and CD96 blocking in BCSCs (Figure 7C and Figure S6F, Supporting Information), suggesting that CD155:CD96 is involved in FAO metabolism. Moreover, there were no significant changes in Opa1 expression in response to a CD96‐blocking antibody in CD155 knockout BCSCs (Figure 7D), indicating that mitochondrial membrane remodeling is involved in the CD155‐CD96 signaling pathway. These results suggest that CD155 is associated with CD96‐mediated BCSCs drug resistance in vitro.

Subsequently, we evaluated whether the CD155:CD96 interaction promotes BCSCs chemoresistance in an immunocompromised mouse model in vivo. NOD/SCID mice were grafted with CD155^+^ and CD155^KO^ BCSCs and then administered docetaxel. In the presence of docetaxel, BCSC‐CD155 knockout largely suppressed tumor growth to the same extent as single CD96‐blocking antibody treatment (Figure 7E). However, CD96 blockage did not further enhance chemosensitivity when CD155 was knocked out (Figure 7E). The first Ig domain of CD96 is the main structure that interacts with CD155 in previous reports.^[^ ^39^, ^50^ ^]^ Among the first Ig domain, the 75 tyrosine are the main amino acid sites for their interaction. To further confirm our hypothesis, we generated a Tyr75Ala variant (75^nd^ tyrosine was mutated to alanine)^[^ ^50^ ^]^ and transfected it in MCF‐7 cells with low CD96 expression. As a negative control, Gln46Ala mutation (46^nd^ Glutamine was mutated to alanine) was included. The apoptosis of Tyr75Ala mutated cells treated with docetaxel is largely increased compared to Gln46Ala mutation in vitro (Figure 7F and Figure S6G, Supporting Information). Consistently, the Tyr75Ala mutation in tumor cells with low CD96 expression promotes chemotherapy efficiency in vivo rather than the Gln46Ala mutation, which was assessed by tumor size and 18F‐flourodeoxyglucose (18F‐FDG) Positron Emission Tomography/Computed Tomography (PET‐CT) (Figure 7G and Figure S6H,I, Supporting Information). Taken together, these findings suggest that the interaction between BCSC‐expressed CD96 and its ligand, CD155, promotes chemoresistance in BC in vivo.

## 3 Discussion

Our study revealed a novel CD155‐CD96‐Src‐Stat3‐Opa1‐dependent mitochondrial membrane remodeling pathway that potentiates FAO metabolism (Figure 7H). This process is critical for regulating the stemness of BCSCs and their associated chemoresistance. Previous studies of CD96 expression focused on its roles in NK cells, T cells, and human acute myeloid leukemia.^[^ ^5^, ^7^, ^11^, ^12^, ^14^, ^15^, ^16^ ^]^ We discovered that there is a subpopulation of CD96^+^ cells in BC and found that their proportion is directly connected to disease progression. The mechanism by which solid tumor‐intrinsic CD96 influences patient survival remains unknown. We demonstrated that CD96 regulates BCSCs and promotes their chemoresistance. Furthermore, the interaction between CD96 and CD155 promotes stemness and chemoresistance in cancer cells by activating Src‐Stat3‐Opa1‐mediated mitochondrial membrane remodeling, which subsequently regulates fatty acid *β*‐oxidation.

Hypermetabolism helps to maintain the stemness and function of CSCs, making it a fundamental target for the development of next‐generation cancer therapies.^[^ ^51^, ^52^, ^53^ ^]^ While hypermetabolism represents a common feature of leukemia stem cells and malignant stem cells from solid tumors, the underlying mechanisms clearly differ probably due to the microenvironment they reside.^[^ ^33^ ^]^ Our study revealed that compared to adherent cells, BCSCs have a higher FAO level, which is consistent with the fact that CSCs primarily rely on FAO‐mediated metabolic processes.^[^ ^27^, ^54^ ^]^ In addition, our data show that CD96 interacts with Src and facilitates phosphorylation of Stat3 in BCSCs, linking this interaction with the regulation of FAO in these cells. These results suggest that CD96‐Src regulates FAO in BCSCs via the Stat3 pathway. However, the mechanism by which this pathway influences FAO metabolism remains unknown. Mitochondria are the main site of FAO in the cell and are tightly regulated via mitochondrial remodeling to help them meet their cellular metabolic demands.^[^ ^55^, ^56^ ^]^ One such remodeling step, mitochondrial fusion, is critical for fatty acid transfer to the mitochondria and subsequent FAO‐mediated regulation of the electron transport chain (ETC) complex associations and oxidative phosphorylation.^[^ ^37^, ^38^ ^]^ Interestingly, we found that anti‐CD96 inhibits Opa1 transcription and mitochondrial fusion, which was rescued by addition of a Stat3 activator. Opa1 is also important for mitochondrial crista maintenance, which is required for effective OXPHOS and FAO. Ablation or alteration of Opa1 leads to disorganization of the cristae and widening of the cristae junctions.^[^ ^57^, ^58^, ^59^, ^60^ ^]^ Moreover, the addition of an FAO agonist reversed the effects of the CD96‐blocking antibody, suggesting that CD96 promotes FAO by stabilizing the mitochondrial cristae junction and maintaining mitochondrial fusion.

The CD96 signaling pathway has remained fairly unclear in solid tumors, although it has been reported to act as a CSC marker in leukemia. The human CD96 protein encodes both inhibition and activation motifs via their ITIM and YXXM domains. This observation is of interest because YXXM can bind SH2 domain‐containing molecules that participate in multiple biological processes, including in cancer^[^ ^61^, ^62^ ^]^ and immune cells.^[^ ^63^, ^64^ ^]^ Comprehensive evaluation of CD96 and its downstream signaling may reveal potential therapeutic benefits. Here, we revealed that CD96 binds to the SH2 domain of Src via the YXXM motif and regulates the Stat3 pathway in BCSCs. P85 may also interact with CD96; however, no experimental data is available to support these interactions.^[^ ^18^ ^]^ Our experiments did not reveal an interaction between CD96 and P85 in BCSCs, suggesting that proteins that bind to the CD96 YXXM motif differ in various cells and require further exploration.

CD96 acts as a receptor for CD155, which activates CD96 and inhibits immune cell function in the tumor microenvironment.^[^ ^7^, ^9^, ^12^ ^]^ Thus, determining the relationship between CD155 and CD96 in BCSCs is important for understanding chemoresistance in these tumors. Our findings were largely consistent with those in previous reports describing marked CD155 expression in both BC cells and BCSCs.^[^ ^65^ ^]^ Interestingly, CD155 directly participates in chemoresistance in cells with high CD96 expression but does not appear to impact this parameter in cells with low CD96 expression. These findings were validated immunocompromised NOD/SCID mice lacking normal NK and T cells. Thus, CD96 is a key regulator of chemoresistance in these cells but must communicate with its ligand, CD155, to exert these effects.

In summary, our findings demonstrate that CD96 is highly expressed in various cancer cell subsets and is associated with malignancy in BC. This expression endows BCSCs with increased chemoresistance via regulation of the FAO pathway. CD96 blocking not only increases anti‐tumor immunity but also promotes tumor cell killing by chemotherapeutic drugs. This BCSC‐CD96 receptor‐driven chemoresistance not only enhances our understanding of the mechanisms of chemoresistance but also will contribute to refining chemotherapy to achieve improved outcomes in patients with cancer.

## 4 Experimental Section

### Patients and Tissue Samples

Tumor samples for survival assay were obtained from invasive breast carcinoma which underwent operation at the Sun Yat‐Sen Memorial Hospital, Sun Yat‐Sen University (Guangzhou, China) between 2000 and 2015. For apoptotic assessment after chemotherapy and some patient‐derived xenograft experiments, patients have accepted neoadjuvant therapy before operation. The chemotherapy regimens were as follows: To HER2‐negative breast cancer, dose‐dense AC (doxorubicin 60 mg m^−2^ plus cyclophosphamide 600 mg m^−2^ every 14 days for 4 cycles), followed by paclitaxel 175 mg m^−2^ every 14 days for 4 cycles; To HER2‐positive breast cancer, doxorubicin 60 mg m^−2^ plus cyclophosphamide 600 mg m^−2^ every 3 weeks for 4 cycles, followed by docetaxel 100 mg m^−2^ every 3 weeks for 4 doses (AC‐T); or docetaxel 75 mg m^−2^ plus carboplatin AUC 6 every 3 weeks for 6 cycles. All related procedures were performed with the approval of the internal review and ethics board of Sun‐Yat‐Sen Memorial Hospital (293). Informed consent was obtained from each participant.

### Primary Tumor Cells Isolation

For the isolation of primary tumor cells, 2 mg mL^−1^ collagenase I, 2 mg mL^−1^ collagenase III, and 2 mg mL^−1^ hyaluronidase were used to obtain single cells from breast cancer tissue, tumor cells were harvested by CD326 (EpCAM) MicroBeads (Cat# 130‐061‐101, Miltenyi) after filtration following the manufacturer's instructions. Cells were used in flow cytometry assay and serial transplantation models.

### Mice

For serial transplantation models, cancer cells were isolated as described in primary tumor cells isolation. Tumor formation was assessed at 6 weeks. For other patient‐derived xenograft (PDXs) experiments, the fresh tumor tissues from clinical breast cancer patients who underwent tumor resection were used within 30–180 min, and a small incision was made on the abdomen of anesthetized NOD/SCID mice and tumor tissues were minced into 1 mm^3^ sized fragments and injected directly into the mammary fat pad. In some experiments, PDXs were injected intraperitoneally with docetaxel (10 mg kg^−1^ per week) in the absence and presence of blocking antibody against CD96 (250 µg per three days, Cat# AA 321‐519, Antibodies‐online) after 6 weeks. Tumor diameter was measured using caliper every 3 days and PET‐CT at 12 weeks for PDXs or 6 weeks for others, therapeutic responses were assessed refer to the human clinical evaluation standard (RECIST). Complete Response (CR) was defined as disappearance of tumor; Partial Response (PR) was defined as at least a 30% reduction in the sum of the longest diameter of target lesions; Progressive Disease (PD) was defined as at least a 20% increase in the sum of the longest diameter of target lesions; and Stable Disease (SD) was defined as neither sufficient shrinkage to qualify as PR nor sufficient increase to qualify as PD. CR and PR were classified as chemosensitivity, while SD and PD were classified as chemoresistance.^[^ ^22^ ^]^

To evaluate the tumor formation ability of BCSCs, tumor cells were transfected with luciferase then enriched to mammospheres, which were serially cultured for 5 weeks to enrich BCSCs.^[^ ^23^, ^66^ ^]^ 5 × 10^4^ BCSCs were injected into the mammary fat pads of 4–6‐week‐old NOD/SCID mice, which were implanted with a 1.7 mg 17b‐estradiol pellet (60‐day release, Innovative Research of America, Sarasota, FL, USA) 3 days before implantation. 10 mg kg^−1^ docetaxel (per week) and 250 µg anti‐CD96 antibodies (every three days) were injected intraperitoneally when tumor was palpable (about 7 days). Tumor volume was measured every 3 days using a caliper and the formula (length × width^2^)/2. Mice were euthanized, and tumor was imaged by Xenogen IVIS Lumina system (Caliper Life Sciences) after giving venously d‐luciferin at 42 days. All related procedures were performed with the approval of the institutional animal care and use committee, Sun Yat‐Sen University (B0769).

### Cell Culture

MCF‐7, SKBR3, MDA‐MB‐231, MDA‐MB‐361, MDA‐MB‐468, BT‐474, BT‐549, and HeLa cells were purchased from American Type Culture Collection (ATCC) and cultured in DMEM medium (Gibco) supplemented with 10% FBS (Gibco) and 100 U mL^−1^ Penicillin–streptomycin (Life Technologies). Human peripheral blood from healthy donors was collected from Guangzhou blood center, T cells were isolated by Ficoll density gradient centrifugation (37 °C, 450×*g*, 20 min, Beckman) and CD3 MiBeads (Cat# 130‐050‐101, Miltenyi), and cultured in 1640 medium (Gibco) supplemented with 10% FBS, 25 U mL^−1^ IL‐2 (Cat# 200‐02, PeproTech) and 100 U mL^−1^ Penicillin–streptomycin (Life Technologies). Mammosphere were enriched and cultured in stem cell media (DMEM/F12 (Gibco), 2% B27 (Life Technologies), 20 ng mL^−1^ hEGF (PeproTech), 20 ng mL^−1^ hFGF2 (PeproTech)). Mammosphere were dissociated with 1 × Tryple Express Enzyme (Cat# 12604021, Life Technologies) for 15 min at 37 °C. Adherent cells were dissociated with 0.25% Trypsin‐EDTA (Thermo).

### Mammosphere Formation and Treatment

Mammosphere formation was conducted in MCF‐7 and SKBR3 cells via stem cell media. One thousand cells were cultured in ultra‐low adhesion plates (Corning), and mammospheres were counted by ImageJ software after 10 days. Mammospheres exceeding 75 µm in diameter were counted as previously reported.^[^ ^67^ ^]^ Cells were transfected with CD96 vector with or without ITIM or YXXM mutation before mammosphere formation assay to explore the influence of CD96 in mammosphere formation. In some experiments, BCSCs were transduced with shRNA against CD96. For all in vitro experiments used CD96 blocking antibody or IgG antibody, 50 µg mL^−1^ antibody was co‐cultured with cells for 2 days before follow‐up experiments. For apoptotic analysis, cell after prolonged mammosphere culture were treated with docetaxel (0, 0.002, 0.02, 0.2, 2.0, 20 µg mL^−1^ for MTT assays, 0.02 µg mL^−1^ for other experiments), cisplatin (0, 10, 20, 30, 40, 50 µm for MTT assays) in the presence and absence of 50 µg mL^−1^ CD96 blocking antibody for 24 h. 5 µm Bezafibrate was added into media with chemotherapy drug to explore the influence of fatty acid *β*‐oxidation (FAO) in BCSCs. In some experiments, cell was treated with 50 µg mL^−1^ anti‐CD96 blocking antibody, Src inhibitor dasatinib^[^ ^68^ ^]^ (100 ng mL^−1^, Cat# S1021, Selleck) or tirbanibulin^[^ ^69^, ^70^ ^]^ (50 nm, Cat# S2700, Selleck) for 24 h for phosphorylation analysis and 10 days for mammosphere formation assay.

### Immunofluorescence or Immunohistochemistry Staining

For immunofluorescence, cells and tissues samples were blocked with 5% BSA for 20 min and incubated with primary antibodies specific for ALDH1 (1:100, Cat# 01‐8340A, American Research Products), CD8 (1:150, Cat# ab17147, Abcam), CD96 (1:150, Cat# PA5‐97568, Thermo) or Cytokeratin (1:100, Cat# MNF116, Abcam) overnight at 4 °C. Samples were incubated with Alexa Fluor‐conjugated secondary antibodies (Invitrogen) for 1 h at room temperature after washing two times. For BODIPY staining, mammospheres were treated as previously described, then dissociated into single cells and attached to coverslip. Cells were fixed in 4% polyformaldehyde for 20 min and stained with 1 µm BODIPY 493/503 (1:1000, Cat# D3922, Thermo) for 30 min. DAPI was then used for counterstain the nuclei and images were obtained by laser scanning confocal microscopy (LSM780 or LSM800, Zeiss).

For immunohistochemistry, tissue samples were treated as described above and incubated with antibodies specific for CD96 (1:50, Cat# PA5‐97568, Thermo), p‐Src (1:100, Cat# 2105, CST), p‐Stat3 (1:100, Cat# 9145s, CST) overnight at 4 °C. The next day, samples were stained with GTVision TMIII immunohistochemistry assay kits (Cat# GK500705, Gene tech) according to the manufacturer's instructions. Nucleus was stained using Hematoxylin. Images were obtained by BX‐63 (Zeiss). The expression levels of CD96 were scored semiquantitatively based on staining intensity and distribution using the immunoreactive score (IRS) as previously described.^[^ ^71^ ^]^ Briefly, Immunoreactive score (IRS) = SI (staining intensity) × PP (percentage of positive cells). SI was assigned as: 0 = negative; 1 = weak; 2 = moderate; 3 = strong. PP is defined as 0 = 0%; 1 = 0–25%; 2 = 25–50%; 3 = 50–75%; 4 = 75–100%. For categorization of the continuous CD96 values into low and high, a commonly used cutoff point for the measurements was chosen.

### Flow Cytometry

Protein expression was evaluated by flow cytometry. Cells were washed two times with phosphate buffered saline (PBS) and suspended in PBS containing 1% FBS, then stained with APC anti‐human CD96 (Cat# 338410, BioLegend), PE anti‐human CD155 (Cat# 337610, BioLegend), APC/Cy7 anti‐human CD24 (Cat# 311131, BioLegend) and Alexa Fluor 488 anti‐human CD44 (Cat# 338830, BioLegend) for 30 min at 4 °C. ALDH1 activity was checked using the ALDEFLUOR kit (Cat# 01705, Stem cell technologies) following the manufacturer's instructions. Briefly, cells were resuspended in 100 µL buffer supplemented with 1 µL ALDH1 substrate with or without 2 µL DEAB at 37 °C for 1 h. CytoFLEX (Backman) was used to check the protein expression or ALDH1 activity of cells after washing two times. Apoptosis was evaluated using an Annexin V Apoptosis Detection Kit (Cat# 640932/640905, BioLegend) according to the manufacturer's instructions. Briefly, cells were mixed with 100 µL binding buffer with 5 µL APC or FITC‐conjugated Annexin V antibody and incubated for 15 min at room temperature. After incubation, the cells were resuspended in binding buffer (200 µL) containing 5 µL of Propidium Iodide and analyzed by CytoFLEX (Backman).

### Western Blot

Proteins were extracted using RIPA buffer (Beyotime Biotechnology) containing protease and phosphatase inhibitor cocktail (Cat# 78446, Thermo) and boiled in 1 × loading buffer, then separated by SDS–polyacrylamide gels and transferred to PVDF membranes. Anti‐CD96 (3 µg mL, Cat# PA5‐97568, Thermo), anti‐Stat3 (1:1000, Cat# ab119352, Abcam), anti‐p‐Stat3 (1:1000, Cat# 4074, CST), anti‐Src (1:1000, Cat# ab109381, Abcam), anti‐p‐Src (1:1000, Cat# sc166860, Santa Cruz Biotechnology), anti‐p85 (1:1000, Cat# 4292, CST), anti‐p‐P85 (1:800, Cat# PA5‐104853, Thermo), anti‐JAK (1:1000, Cat# 3344, CST), anti‐p‐JAK (1:1000, Cat# 3771, CST), anti‐Opa1 (1:1500, Cat# PA5‐98029, Thermo), anti‐CD155 (1:1000,Cat # PA5‐96414, Thermo), or anti‐GAPDH (1:5000, Cat# 8884,CST) was diluted in TBST containing 5% BSA and incubated with membranes overnight at 4 °C. Peroxidase‐conjugated secondary antibody (1:3000, Cat# 7074 or Cat# 7076, CST) was co‐cultured with membranes and the antigen–antibody reactions were visualized with enhanced chemiluminescence assays (ECL, Thermo).

### Plasmids and shRNA Oligonucleotides

The medium was replaced before transduction for 1 h. ShRNA against CD96, Src, Stat3, Opa1, or GFP (as a control) was transduced into single cells by 5 × 10^7^ lentiviral particles (multiplicity of infection of 10, Genepharma) supplemented with 8 µg mL^−1^ Polybrene (Sigma) for 8–12 h. The silenced efficiency was evaluated by western blot after transduction 2 days. For plasmid transfection, opti‐MEN medium was mixed with plasmid (Genepharma, Shanghai) and Lipofectamine 3000 (Cat# L3000015, Thermo) respectively. LP‐300 was added at a 1:2 ratio to the plasmid, which was added into cells after 5 min. Cells were incubated for 12 h and the medium was changed. Protein expression was detected by western blotting after 2 days. The sequences as follow:

Sh‐GFP: TAGCGACTAAACACATCAA;

Sh‐CD96‐1: CCAACGAAAGTGATCTGCC;

Sh‐CD96‐2: AGTGGAAGGTACGAGTGTA;

Sh‐Src‐1: GCTCGGCTCATTGAAGACA;

Sh‐Src‐2: GACAGACCTGTCCTTCAAG;

Sh‐Stat3‐1; GCAAAGAATCACATGCCAC;

Sh‐Stat3‐2; GCACAATCTACGAAGAATC;

Sh‐Opa1‐1: GCCTGACATTGTGTGGGAAAT;

Sh‐Opa1‐2: GCTCCTGACACAAAGGAAACT;

### MTT Assay

1 × 10^3^ Cells were seeded onto 96‐well plates and incubated overnight at 37 °C. Next day, cells were treated with the indicated agents for 24 h.^[^ ^72^ ^]^ 5 mg mL^−1^ 3‐(4,5‐dimethylthiazol‐2‐yl) 22,5‐diphenyltetrazolium bromide (MTT, Sigma) was added and incubated for 4 h at 37 °C. After centrifugation (500 × *g*, 5 min), a supernatant was removed carefully. 150 µL DMSO was added and mixed to dissolve the formazan crystals. The absorbance was measured at 540 nm by Infinite F500 (Tecan).

### PET‐CT

Mice were fasted for 8 h and then anesthetized with pentobarbital. 18F‐FDG (5 ci g^−1^) in 100 µL saline was injected via the tail vein, static scan was performed after injection 20–30 min with an Inveon micro‐PET‐CT Scanner (Siemens, Germany). The micro PET images were corrected for attenuation, scatter, normalization, and camera dead time and co‐registered with micro CT images. The tumor uptake of 18F‐FDG was calculated in terms of the standardized uptake value (SUV) in three‐dimensional regions of interest.

### IVIS Lumina Imaging

IVIS images were obtained as per previous report.^[^ ^73^ ^]^ Briefly, NOD‐SCID mice were injected with MCF‐7 BCSCs pre‐transduced with lentivirus carrying luciferase expressing plasmid (Genepharma, Shanghai) to examine the growth of tumor cells in mice through IVIS lumina imaging. Mice were given d‐luciferin (300 mg kg^−1^ i.v., 10 min before imaging), anesthetized (3% isoflurane), and imaged with a Xenogen IVIS Lumina system (Caliper Life Sciences). Images were analyzed with Living Image software v. 3.0 (Caliper Life Sciences). Bioluminescent flux (photons s^−1^ cm^−2^ sr^−1^) was determined for tumor growth.

### CD96 and TUNEL Staining

Tumor tissues were cut into 4‐µm‐thick sections and then mounted on glass slides. The slices underwent dewaxing, hydration and antigen retrieval for 15 min in 0.01 m citrate buffer (pH 6.0). Then, samples were blocked in PBS containing 5% bovine serum albumin (BSA) for 20 min at room temperature. CD96 antibody was co‐incubated with samples overnight at 4 °C. The next day, samples were stained using in situ Cell Death Detection Kit (Cat# 11684817910, Roche) at 37 °C for 30 min. For cells apoptotic analysis using TUNEL staining, adherent BC cells and BCSCs were dissociated with 0.25% Trypsin‐EDTA or 1 × Tryple Express Enzyme, respectively. Cells were stained using in situ Cell Death Detection Kit as above described. DAPI was used for counterstain of the nuclei. Images were obtained by laser scanning confocal microscopy (LSM780 or LSM800, Zeiss).

### Site‐Directed CD96 Mutagenesis and Enforced Expression in Cells

The ITIM and YXXM motif of CD96 were mutated to phenylalanine using the GENEART Site‐Directed Mutagenesis System (Invitrogen) according to the manufacturer's protocols. The mutagenic primers as follow:

Human CD96 ITIM mutation site (Y566F):5’‐CACCACCTCCCATCAAGTTTACT

TGCATTCAAGAGCCC‐3’;

Human CD96 YXXM mutation site (Y579F): 5’‐AACGAAAGTGATCTGCCTTTT

CATGAGATGGAGACCCTC‐3’;

To assessed the interaction between CD96 and CD155, Tyr75Ala (75^nd^ tyrosine was mutated to alanine) and Gln46Ala (46^nd^ Glutamine was mutated to alanine) variants were generated, respectively. The mutagenic primers as follow:

Tyr75Ala variant: GCTGTCTATCATCCCCAAGCTGGCTTCTACTGTGCCTAT;

Gln46Ala variant: GATGTCAACCTGACCTGCGCTACACAGACAGTAGGCTTC;

Fidelity of vectors was validated by bidirectional sequencing using the human CD96 cloning primers.

Human CD96 (forward): TTCCTCAACAGACCCTCCAC;

Human CD96 (reverse): TGGGTTGAGGAGTGGTGTTT;

Mutant CD96 variants were inserted into the EcoRI/NotI sites of pcDNA3.1‐6his plasmids and transfected into indicated cells. Wild‐type or mutant CD96 overexpression was confirmed by western blotting.

### Immunoprecipitation

HeLa cells were transfected with the mutant or wild‐type CD96 and Src plasmids for 48 h, then the cells were lysed in IP lysis buffer (Cat# 87787, Thermo Scientific) with Protease and Phosphatase Inhibitor Cocktail (Cat# 78446, Thermo Scientific). Lysates were incubated with the anti‐CD96 (1:50, Cat# PA5‐97568, Thermo) for 1 h. 50 µL of Dynabeads Protein A (Cat# 10001D, Thermo Scientific) was added for 1 h at room temperature. The protein complex was washed three times with IP lysis buffer, eluted with 1 × loading buffer by boiling for 5 min and resolved by 10% SDS‐PAGE followed by immunoblotting with the CD96 or Src antibodies.

### Seahorse Metabolic Analyzer

Seahorse experiments was performed as previously reported.^[^ ^35^ ^]^ Briefly, 10 000 cells per well were seeded in Seahorse XF24 cell culture plate overnight. Next day, cells were treated with 100 ng mL^−1^ dasatinib, 50 µg mL^−1^ IgG or anti‐CD96 for 24 h. oxygen consumption rate (OCR) measurements were taken using Seahorse XFe24 analyzer (Agilent) according to the manufacturer's protocol. Culture media was replaced with FAO assay medium (111 mm NaCl, 4.7 mm KCl, 1.25 mm CaCl_2_, 2 mm MgSO_4_, 1.2 mm NaH_2_PO_4_, supplemented with 2.5 mm Glucose, 0.5 mm carnitine and 5 m, pH 7.4) before 1 h. Palmitate‐BSA (PLMA, Cat# 102720‐100, Seahorse Bioscience) was applied at a final concentration of 0.1 mm just before the start of the assay. 40 mm Etomoxir, 4 mm Oligomycin, 2 mm FCCP, 2 mm Antimycin A were used during OCR measurement.

### Fatty Acid Assay

Free fatty acid (FFA) levels in adherent BC cells and BCSCs were measured using the Free Fatty Acid Quantification Kit (Cat# ab65341, Abcam) according to the manufacturer's instructions. Briefly, 10^6^ cells were harvested and homogenized in 200 µL chloroform/Triton X‐100 (1%Triton X‐100 in pure chloroform). Samples were incubated on ice 30 min and Spined 10 min. Lower phase was collected and performed with air dry at 50 °C and vacuum dry, respectively, to remove trace chloroform. Dissolving the dried lipids in 200 µL of Fatty Acid Assay Buffer by vortex extensively for 5 min. Samples were mixed with assay buffer, fatty acid probe, enzyme mix and enhancer at 37 °C for 30 min protected from light. Output was immediately measured by a microplate reader at OD 570 nm.

### Quantitative PCR with Reverse Transcription (qRT–PCR)

Total RNA was extracted with TRIzol Reagent (Cat# 10296010, Thermo) according to standard protocols. RNA was reverse‐transcribed into cDNA using the PrimeScript RT reagent Kit (Cat# RR037B, Takara), then preformed qRT–PCR analysis using TB Green Advantage qPCR premixes (Cat# 639676, Takara). A LightCycler 480 instrument (Roche) was used to collect and analyze data. The primer sequences as follow:

Opa1 (Forward): GGAATGACTTTGCGGAGGAC;

Opa1 (Reverse): ACACTGTTCTTGGGTCCGAT;

Mfn1 (Forward): TGTTTTGGTCGCAAACTCTG;

Mfn1 (Reverse): CTTTGAGCTCCTCCACCAAG;

Mfn2 (Forward): CATGGGCATTCTTGTTGTTG;

Mfn2 (Reverse): TGGAGCCAGTGTAGCTGATG;

Drp1 (Forward): CAGTGTGCCAAAGGCAGTAA;

Drp1 (Reverse): GATGAGTCTCCCGGATTTCA;

Mff (Forward): TGGACAGCTGGTCAGAAATG;

Mff (Reverse): ATCTGCTGGTATGCCCTACG;

### Electron Microscopy

The crista of mitochondria was evaluated by transmission electron microscopy as previously reported.^[^ ^74^ ^]^ In brief, cells were fixed and dehydrated through a graded ethanol series and placed in embedding boards to which epoxy resin was added. The samples were polymerized at 60 °C for 48 h. Ultrathin sections were cut at a thickness of 70 nm and collected on naked copper‐mesh grids. The grids were stained with uranyl acetate and lead citrate and examined by transmission electron microscopy (Tecnai G2 Spirit BioTWIN). Mitochondria were delineated using ImageJ software for determination of mitochondrial surface and average size (total mitochondria surface area/number of mitochondria) as previously reported.^[^ ^75^ ^]^

### CRISPR‐Mediated Gene Knockout

The sequences targeting CD155 were gRNA (5′‐GTCACAGCTGACTTGGGCG‐3′). The Cas9 lentivirus and gRNA lentivirus were purchased from GenePharma and transduced to MCF‐7 cells. The transduced cells were selected with 2.5 µg mL^−1^ puromycin for 2 weeks to obtain the CD155 knockout MCF‐7 cells, then BCSCs were enriched as previously mentioned.

### Statistical Analysis

Statistical analyses were processed using Graph‐pad prism 7 software. Statistical analysis of each experiment was performed at least 3 times. Two tailed Student's *t*‐test or One‐Way ANOVA were used to detect differences between two groups or more than two groups, respectively. X‐tile statistical software was used to determine an optimal cutoff point by a minimal *p* value approach as previously reported. Multivariable Cox regression analysis was used to determine the independent prognostic factors. Kaplan–Meier survival curves were plotted and the log‐rank (Mantel–Cox) test was used to compare survival curves. Fisher's exact test was used in the serial transplantation models. Spearman correlation assay was used to estimate the correlation between CD96 expression and clinicopathologic features. All data are shown as Mean ± SD (Standard Deviation).

## Conflict of Interest

The authors declare no conflict of interest.

## Author Contributions

J.L., Q.X., and C.D. contributed equally to this work. Q.Z. and J.L. conceived the ideas and designed the experiments. J.L., Q.X., C.D., C.L., H.S., B.Z., S.Y., Y.H.L., and J.H. performed the experiments. J.L., Q.X., C.D., Y.W.L., M.H., H.L., and X.L analyzed the data. Q.Z. and J.L. wrote the paper.

## Supporting information

Supporting Information

## Data Availability

The data that support the findings of this study are available from the corresponding author upon reasonable request.
