# Supplementary material for: Tumor Cell‐Intrinsic CD96 Mediates Chemoresistance and Cancer Stemness by Regulating Mitochondrial Fatty Acid β‐Oxidation
Source: Adv Sci (Weinh). 2022 Dec 29;10(7):2202956. doi: 10.1002/advs.202202956 (PMC9982582; doi:10.1002/advs.202202956)
Supplement: Supplementary file 1 — Supporting Information [file ADVS-10-2202956-s001.pdf]

ADVANCED SCIENCE
Open Access

Supporting Information

for *Adv. Sci.*, DOI 10.1002/advs.202202956

Tumor Cell-Intrinsic CD96 Mediates Chemoresistance and Cancer Stemness by Regulating Mitochondrial Fatty Acid β-Oxidation

*Jiang Li, Qidong Xia, Can Di, Chunni Li, Hang Si, Boxuan Zhou, Shubin Yu, Yihong Li, Jingying Huang, Yiwen Lu, Min Huang, Huixin Liang, Xinwei Liu and Qiyi Zhao**

Figure S1

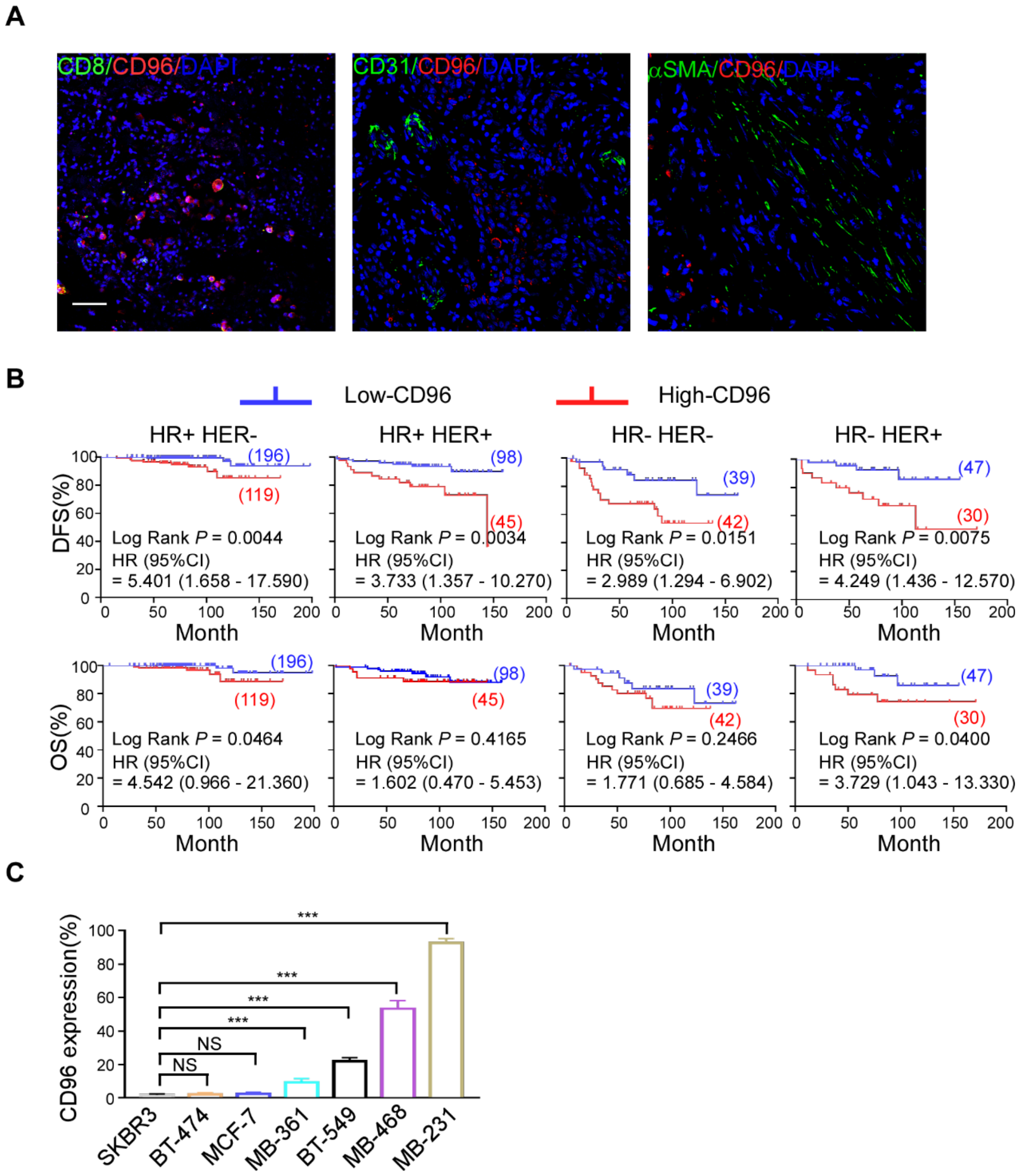

Figure S1. $CD96^+$ cancer cell subsets are associated with poor prognosis and chemoresistance in breast cancer patients. A) Representative immunofluorescence staining of CD96 (red), CD8, CD31 and α-SMA (green) in breast cancer samples. Nuclei were counterstained with DAPI (blue). Scale bars, 50 µm. B) Kaplan-Meier survival curves for patients with breast cancer containing low and high CD96 abundance in different subtypes (ER^+HER^-, ER^+HER^+, ER^-HER^- and ER^-HER^+) of

breast cancers (n = 616). C) CD96 expression was detected by flow cytometry in indicated breast cancer cell lines. Significance was determined by two tailed student's t-test. Mean ± SD; NS > 0.05; ***$P < 0.001$ compared to SKBR3, n = 5.

Figure S2

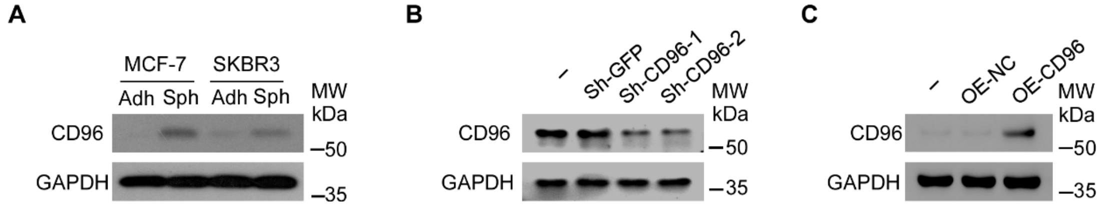

Figure S2. CD96 is highly expressed in BCSCs. A) CD96 expression was detected by western blot in adherent cells (Adh) and stem-like cell mammospheres (Sph) of MCF-7 and SKBR3. B) CD96 was silenced by shRNA in SKBR3 BCSCs and the efficiency was detected via immunoblot. C) CD96 protein was overexpressed in MCF-7 cells and the efficiency was detected via immunoblot. The representative blot is shown from 3 independent experiments in A - C.

Figure S3

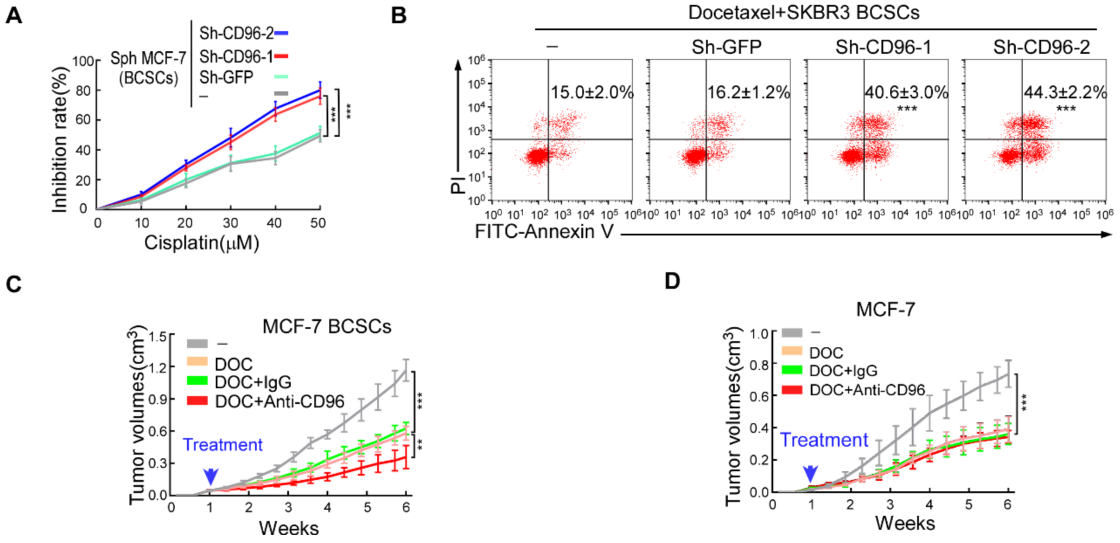

Figure S3. BCSCs-expressed CD96 enhances chemoresistance and promotes tumor progression. A) The growth inhibition rates of cisplatin in MCF-7 BCSCs with CD96 knockdown. Mean ± SD; ****P* < 0.001 compared to BCSCs alone by One-Way ANOVA with Tukey's multiple comparisons test (n = 4). B) SKBR3 BCSCs with CD96 knockdown were treated with docetaxel 24 hr. The proportion of Annexin V$^+$/PI$^-$ (early apoptosis) and Annexin V$^+$/PI$^+$ (late apoptosis) cells were detected by flow cytometry. Mean ± SD; ****P* < 0.001 compared to BCSCs alone by One-Way ANOVA with Tukey's multiple comparisons test (n = 3). C, D) MCF-7 BCSCs or MCF-7 were transplanted into NOD/SCID mice, docetaxel (DOC) and CD96 blocking antibody were injected intraperitoneally when tumor was palpable. Tumor size was evaluated every 3 days, arrow indicates the beginning of treatment when tumor was palpable. ***P* < 0.01; ****P* < 0.001 by One-Way ANOVA with Tukey's multiple comparisons test at week 6, n = 5/group.

Figure S4

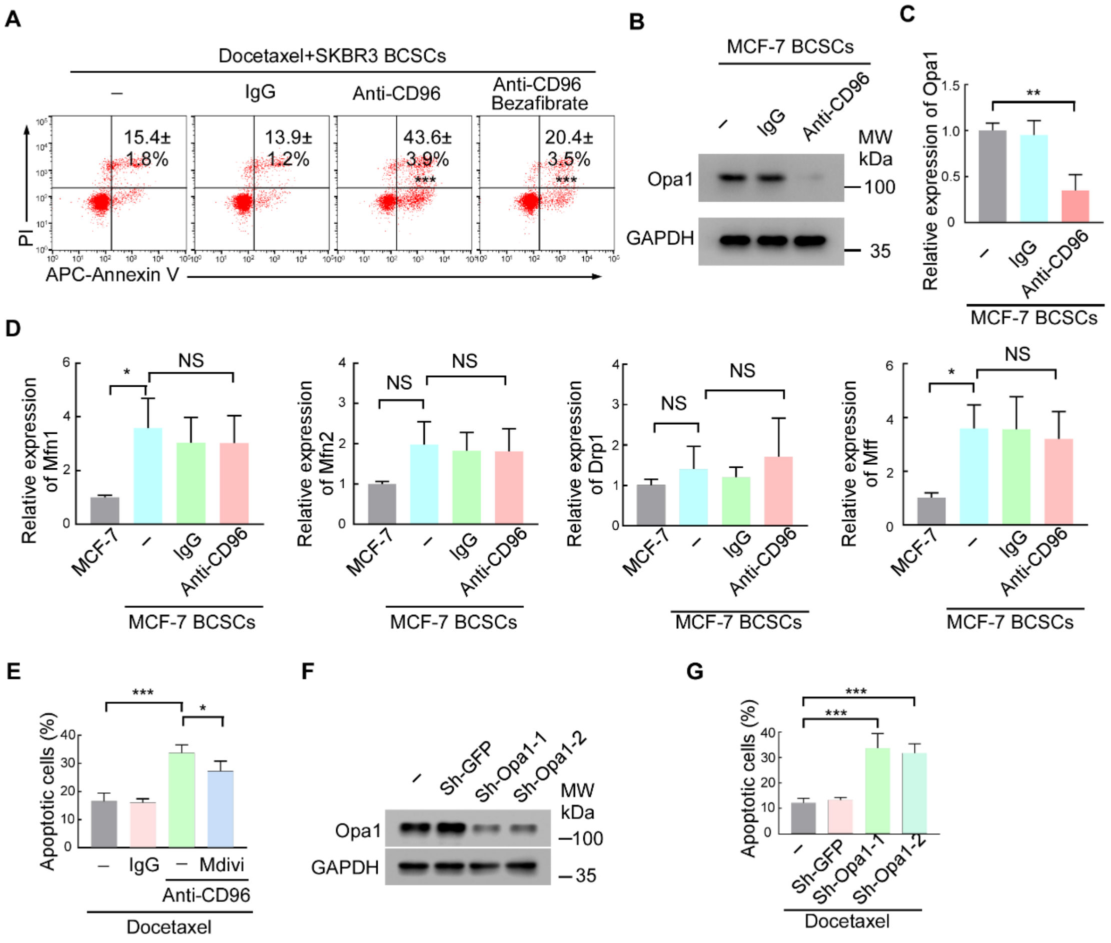

Figure S4. CD96 regulates BCSCs chemoresistance via regulating mitochondrial membrane remodeling and FAO. A) SKBR3 BCSCs were treated with docetaxel and CD96 blocking antibody in the presence and absence of FAO agonists (bezafibrate). The proportion of Annexin V^+/PI^- (early apoptosis) and Annexin V^+/PI^+ (late apoptosis) cells were detected by flow cytometry. The representative plots and quantification are shown. B-D) MCF-7 BCSCs were treated with CD96 blocking antibody with or without FAO agonists. Western blotting of Opa1 protein is shown from 3 independent experiments (B). Opa1, Mfn1, Mfn2, Drp1 and Mff mRNA were checked by qRT-PCR and the relative levels are shown (C, D). E) MCF-7 BCSCs were treated with docetaxel and CD96 blocking antibody with or without

mitochondrial fission inhibitor (Mdivi). Apoptosis cells were checked by flow cytometry and the quantification is shown. F) Opa1 gene was silenced by shRNA and the efficiency was detected via immunoblot. The representative blot is shown from 3 experiments. G) The quantification of apoptosis cells in MCF-7 BCSCs with Opa1 knockdown after treatment with CD96 blocking antibody and docetaxel. Mean ± SD; NS > 0.05; $*P < 0.05$; $**P < 0.01$; $***P < 0.001$ by One-Way ANOVA with Tukey's multiple comparisons test, n = 3 for A, C, D. n = 4 for E and G.

Figure S5

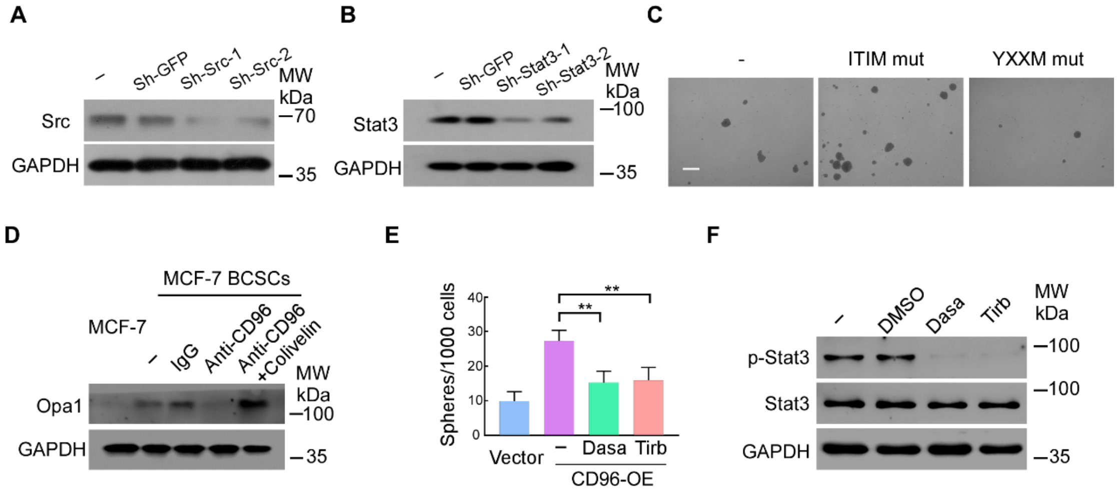

Figure S5. CD96 regulates mitochondrial FAO via the Src-Stat3 pathway in BCSCs. A,B) Src and Stat3 genes were silenced by shRNA and the efficiency was detected via immunoblot. The representative blot is shown (n = 3). C) MCF-7 cells were transfected with CD96 expression vector with ITIM or YXXM mutation. "-" expresses no treatment. The mammosphere forming is shown. Scale bar, 200 μm (n = 3). D) MCF-7 BCSCs were treated with CD96 blocking antibody with or without Stat3 promoter (Colivelin). Representative western blotting of Opa1 protein is shown from 3 independent experiments. E) The quantification of sphere formation in Figure 6J. 4 fields/samples; Mean ± SD; $^{**}P < 0.01$ by One-Way ANOVA with Dunnett-t test (n = 3). F) Total and phosphorylation of Stat3 was detected by western blotting in BCSCs in the presence of Src inhibitor (Dasatinib and Tirbanibulin). The representative blot is shown (n = 3).

Figure S6

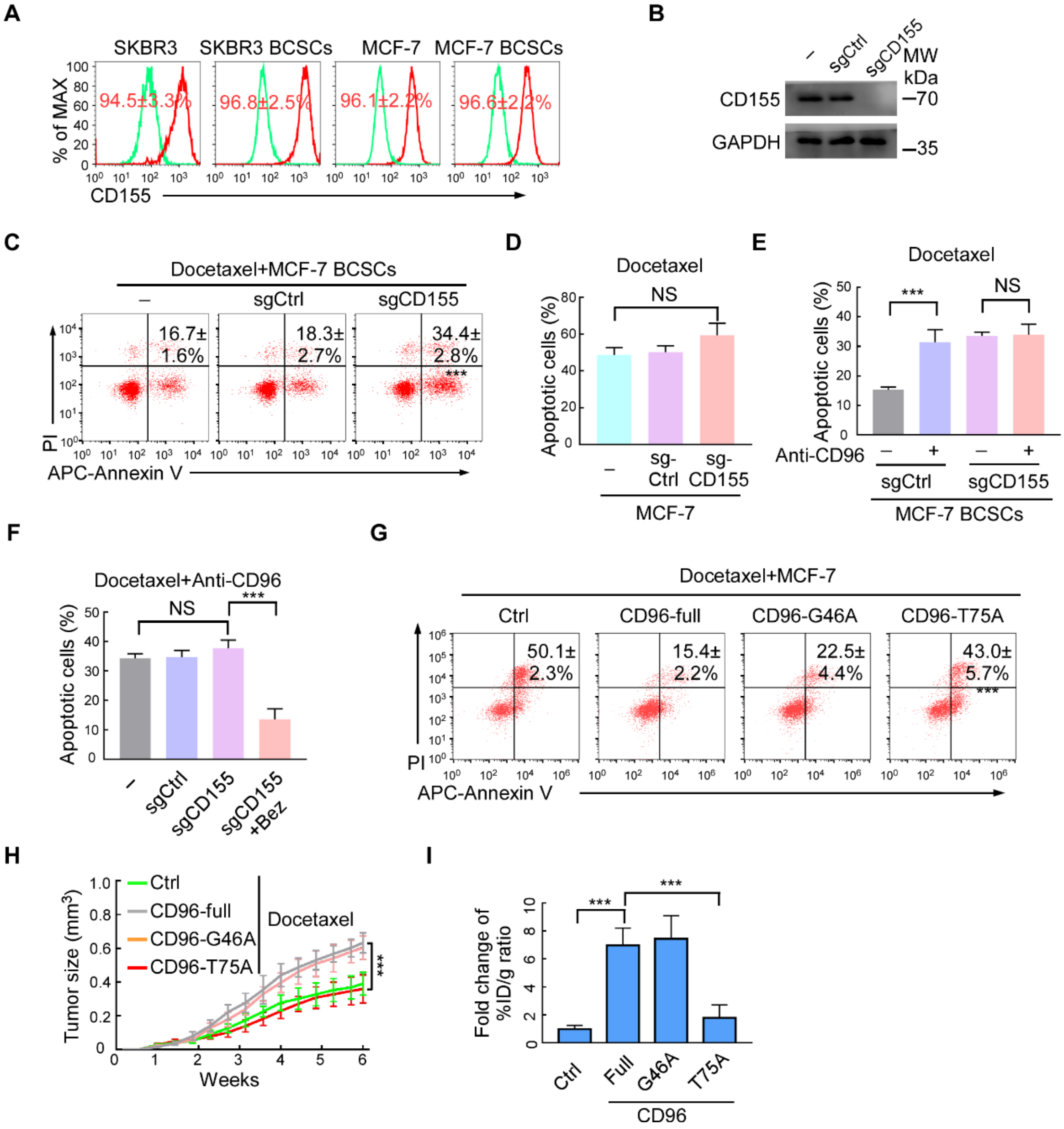

Figure S6. CD96 mediates chemoresistance in BCSCs requires CD155. A) CD155 expression of SKBR3 and MCF-7 cells and BCSCs was detected via flow cytometry. B) Western blotting for CD155 expression in MCF-7 BCSCs that were untreated (-), transfected with a control sgRNA (sgCtrl) or with CD155 sgRNA (n = 3). C, D) MCF-7 BCSCs and MCF-7 with CD155 knockout were treated with docetaxel. Apoptosis cells were checked by flow cytometry. The representative plots and quantification of apoptosis cells are shown (n =3). E) The quantitation in Figure 7B is

shown. F) The quantitation in Figure 7C is shown. G-I) MCF-7 cells infected with CD96-full and indicated mutation plasmid and treated with docetaxel in vitro and vivo. G) The proportion of Annexin V^+/PI^- (early apoptosis) and Annexin V^+/PI^+ (late apoptosis) cells were detected by flow cytometry. The quantitation is shown. H) Cells were transplanted into NOD/SCID mice. Docetaxel was injected intraperitoneally when tumor was palpable. Tumor size was evaluated every 3 days. I) The relative quantitation in Figure 7G is shown. Percent ID, percent injected dose. Mean ± SD; NS > 0.05; ***p < 0.001 by One-Way ANOVA with Tukey's multiple comparisons test exclude E (two tailed Student's t-test), n = 4 for A, n = 3 for C-G, n = 5/group for H,I at week 6.

Table S1. Clinical features of patients with breast cancer.

Factor	N	Percentage (%)
**Age group**		
≤45	252	40.9%
>45	364	59.1%
**Menstruation status**		
Premenopause	317	51.5%
Menopause	299	48.5%
**Tumor size**		
≤2cm	362	58.8%
>2cm	254	41.2%
**Lymph node**		
Negative	369	59.9%
Positive	247	40.1%
**Histological grade**		
I/II	357	58.0%
III	259	42.0%
**Molecular subtype**		
$HR^{+}HER2^{-}$	315	51.1%
$HR^{+}HER2^{+}$	143	23.2%
$HR^{-}HER2^{-}$	81	13.2%
$HR^{-}HER2^{+}$	77	12.5%

Abbreviations: HR, hormone receptor; HER2, human epidermal growth factor receptor.

Table S2. Cox regression analysis of OS in patients with breast cancer.

Overall Survival				
	Univariate	Multivariate		
	P	HR	95%CI	*P*
Age (>45)	0.107			
Premenopausis	0.289			
Tumor size (>2cm)	<0.001*	2.303	1.247-4.254	0.008*
Lymph node (positive)	<0.001*	3.363	1.794-6.305	<0.001*
Grade (III)	0.265			
ER (positive)	<0.001*	0.341	0.147-0.791	0.012*
PR (positive)	<0.001*	0.605	0.264-1.386	0.235
HER2 (positive)	0.09			
CD96 expression (high)	0.002*	2.265	1.241-4.135	0.008*

The Univariate and Multivariate Cox values of OS in Age, Premenopausis, Tumor size, Lymph node, Grade, Molecular subtyping and CD96 expression. CD96 is an independent prognostic factor for OS after adjusting for other prognostic variables (n = 616). * represents statistically significant. Abbreviations: PR, progesterone receptor; ER, estrogen receptor; HER2, human epidermal growth factor receptor.

Table S3. Cox regression analysis of DFS in patients with breast cancer.

	Disease-Free Survival			
	Univariate	Multivariate		
	P	HR	95%CI	*P*
Age (>45)	0.507			
Premenopausis	0.783			
Tumor size (>2cm)	<0.001*	2.682	1.567-4.592	<0.001*
Lymph node (positive)	<0.001*	3.635	2.122-6.228	<0.001*
Grade (III)	0.002*	0.898	0.532-1.516	0.687
ER (positive)	<0.001*	0.326	0.152-0.697	0.004*
PR (positive)	0.001*	0.794	0.371-1.700	0.552
HER2 (positive)	0.033*	1.623	0.990-2.661	0.055
CD96 expression (high)	<0.001*	3.915	2.264-6.772	<0.001*

The Univariate and Multivariate Cox values of DFS in Age, Premenopausis, Tumor size, Lymph node, Grade, Molecular subtyping and CD96 expression. CD96 is an independent prognostic factor for DFS after adjusting for other prognostic variables (n = 616). * represents statistically significant. Abbreviations: PR, progesterone receptor; ER, estrogen receptor; HER2, human epidermal growth factor receptor.
